# Personal

**Published:** 1895-04

**Authors:** 


### Personal.

DR. CHARLES A. L. REED, of Cincinnati, announces the removal, during March (1895), of his private hospital for abdominal and pelvic surgery from 487 West Sixth street to his recently acquired property in St. Leger place, Walnut hills. The situation is salubrious, quiet, high and possesses a commanding view, while the hospital itself fulfils all modern requirements.—*Mississippi Medical Monthly.*

---

DR. W. N. WISHARD, of Indianapolis, president of the Mississippi Valley medical association, recently visited Buffalo on business connected with the affairs of the association, which holds its next meeting in Detroit, beginning the first Tuesday of Sepember, 1895.

Dr. Wishard has gone east and will spend a few days in New York and Philadelphia before his return home.

---

DR. H. W. QUIRK, of Cleveland, O., says the *Cleveland Medical Gazette*, has returned from Europe, whither he has been for observation and study during the past several months.

---

DR. BERNARD BARTOW, of Buffalo, has removed from 220 Franklin street to 481 Delaware avenue, where the practice of his specialty, orthopedic surgery, will be continued.

---

DR. EDWARD CLARK, of Buffalo, has removed from 271 Franklin street to 378 Franklin street, where he will continue the practice of his specialty, diseases of the rectum.

---

DR. JULIUS POHLMAN, of Buffalo, has removed his office from 367 to 382 Franklin street. He is located in the Cutler building, between Tupper and Edward streets, where he will still devote himself to the practice of ophthalmology.

